# Toward an interactive article: integrating journals and biological databases

**DOI:** 10.1186/1471-2105-12-175

**Published:** 2011-05-19

**Authors:** Arun Rangarajan, Tim Schedl, Karen Yook, Juancarlos Chan, Stephen Haenel, Lolly Otis, Sharon Faelten, Tracey DePellegrin-Connelly, Ruth Isaacson, Marek S Skrzypek, Steven J Marygold, Raymund Stefancsik, J Michael Cherry, Paul W Sternberg, Hans-Michael Müller

**Affiliations:** 1 Division of Biology, California Institute of Technology, Pasadena, CA 91125, USA; 2 Department of Genetics, Washington University School of Medicine, Saint Louis, MO 63110, USA; 3 Dartmouth Journal Services, Pilgrim Five, Suite 5, 5 Pilgrim Park Road, Waterbury, VT 05676, USA; 4 Genetics Society of America, 9650 Rockville Pike, Bethesda, MD 20814-3998, USA; 5 Department of Genetics, Stanford University School of Medicine, Stanford, CA 94305, USA; 6 Howard Hughes Medical Institute, 4000 Jones Bridge Road, Chevy Chase, MD 20815, USA; 7 Department of Genetics, University of Cambridge, Downing Street, Cambridge, CB2 3EH, UK

## Abstract

**Background:**

Journal articles and databases are two major modes of communication in the biological sciences, and thus integrating these critical resources is of urgent importance to increase the pace of discovery. Projects focused on bridging the gap between journals and databases have been on the rise over the last five years and have resulted in the development of automated tools that can recognize entities within a document and link those entities to a relevant database. Unfortunately, automated tools cannot resolve ambiguities that arise from one term being used to signify entities that are quite distinct from one another. Instead, resolving these ambiguities requires some manual oversight. Finding the right balance between the speed and portability of automation and the accuracy and flexibility of manual effort is a crucial goal to making text markup a successful venture.

**Results:**

We have established a journal article mark-up pipeline that links GENETICS journal articles and the model organism database (MOD) WormBase. This pipeline uses a lexicon built with entities from the database as a first step. The entity markup pipeline results in links from over nine classes of objects including genes, proteins, alleles, phenotypes and anatomical terms. New entities and ambiguities are discovered and resolved by a database curator through a manual quality control (QC) step, along with help from authors via a web form that is provided to them by the journal. New entities discovered through this pipeline are immediately sent to an appropriate curator at the database. Ambiguous entities that do not automatically resolve to one link are resolved by hand ensuring an accurate link. This pipeline has been extended to other databases, namely Saccharomyces Genome Database (SGD) and FlyBase, and has been implemented in marking up a paper with links to multiple databases.

**Conclusions:**

Our semi-automated pipeline hyperlinks articles published in GENETICS to model organism databases such as WormBase. Our pipeline results in interactive articles that are data rich with high accuracy. The use of a manual quality control step sets this pipeline apart from other hyperlinking tools and results in benefits to authors, journals, readers and databases.

## Background

The development of linking tools using automatic entity recognition is an active area of research. One such recent tool is *Reflect *[1]. *Reflect *identifies and highlights gene names, protein names and small molecules. When a user clicks on a highlighted entity in a *Reflect*-processed article, a pop-up window displays relevant information about the entity mined from a core source of databases. *Reflect *can be invoked from within a web browser by the use of a browser plug-in making this hyperlinking tool quite portable, and as a fully automated hyperlinking tool, it is fast. However, the downside of being fully automated is that it might lack accuracy and depth.

Automated tools have become excellent at identifying entities that exist in a database, but are necessarily limited in accuracy to those entities that are unique i.e., they are not ambiguous within or across databases. Further, since automated tools only identify entities that already exist in a database, they miss the most relevant information in a research paper - the entity that has been discovered but has yet to be entered into a database. Finally, several of these tools are limited in scope to a minimum number of entity types e.g., genes, proteins, or chemicals; whereas understanding the science in a research paper often requires a reader to understand more about the research system in which these genes and proteins are assayed and concluded to work. Such information, if not presented in the paper leaves novice scientists and scientists outside of the field of study at the mercy of the authors' writing ability and journal-imposed word limits; and whereas this information, such as species specific anatomy and phenotypes are available in databases, access to the relevant pages in the database may not be inherently obvious to the non-initiated database user.

Here we describe a pipeline by which interactive full-text HTML/PDF journal articles are published with named entities in articles linked to corresponding resource pages in biological databases. Such interactive articles allow a reader to click on a gene, protein, transgene, or potentially any object found in the database, and direct the reader to the relevant webpage. This seamless connection from the article to summaries of single-dimensional and high-dimensional data types promotes a deeper level of understanding for the naïve reader and incisive evaluation for the sophisticated reader. Furthermore, this immediate connection to primary data advances planning and conducting the next generation of investigations. Finally, ongoing curation at the biological database ensures that linked content evolves with the field. This linking has already proved successful in the result pages of the Textpresso search engine [2], developed by some of the authors of this paper [3].

The linking pipeline explained in this article has been established for articles discussing the model organism *Caenorhabditis elegans *(*C. elegans*) published in the journal GENETICS. The databases used for this project are WormBase [4] for linking *C. elegans *articles and Saccharomyces Genome Database (SGD) [5] for linking *Saccharomyces cerevisiae *(*S. cerevisiae*) articles. Further, a pipeline is under construction with Flybase [6] for linking *Drosophila melanogaster *(*D. melanogaster*) articles.

## Participating members

### Journal and journal services

In 2009, editors of the journal GENETICS [7] approached WormBase to provide linking from *C. elegans *articles to WormBase. GENETICS articles are processed by Dartmouth Journal Services (DJS). DJS provides XML formatted files for linking through a web service to WormBase. After linked files undergo a manual quality control step at WormBase, the linked file is returned to DJS. DJS processes the linked file creating a linked PDF, which is sent back to WormBase for final approval and then to the author for a final proof. If mistakes are found during the PDF proof stage they are fixed by DJS. Questions concerning ambiguities and formatting errors that arise during the quality control step at the database are relayed to the author through DJS.

### Authors

**Figure 1:**
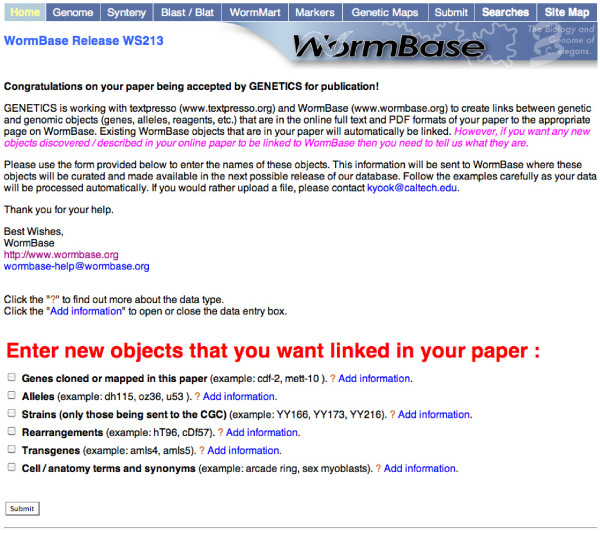
**Author form**. This form helps authors declare any new entities they have discovered or described in their article.

WormBase provides a web form for the authors (Figure 1) to declare entities they have discovered or described, which do not yet exist in the database. A link to this web form is emailed to authors by the journal as soon as their article is accepted for publication. The form itself should take authors only a matter of minutes to understand and complete. Data entered through the form are added directly to the database-specific lexicon for linking, and thus needs to be machine readable; therefore, data submitted through the form is monitored by a WormBase curator to make sure the data are formatted correctly for the pipeline.

If any new objects declared through the author form are not in WormBase at the time of linking, the links for the new, author-declared entities are silent when they are inserted into the article (prior to actual publication). The author form also alerts WormBase curators to the presence of these new entities, which will then be added to the next release of the database by the curator. Links to these new entities are usually live when the article is published or soon thereafter.

### Databases

In this pipeline, databases are required to provide entity lists for linking, a URL pattern for the link, a curator for manual quality control and support for URL stability. For *C. elegans *articles, the database WormBase provided entity lists for the following eleven classes: Anatomy terms, Author names, Clones, Genes, Phenotypes, Proteins (linked to Gene pages), Rearrangements, Sequences, Strains, Transgenes and Variations. Scripts run daily to query the database to keep the entity lists up to date. The URL pattern for linking almost all entities is http://www.wormbase.org/db/get?name=NAME;class=CLASS, where NAME is the entity string (eg: *lin-11, ctDf2, e189*) and CLASS is the class the entity belongs to (*Gene, Rearrangement, Variation*, respectively, see Table 1). For 'Author names' we need an ID for the article and the link is formed as http://www.wormbase.org/db/misc/person?name=NAME;paper=ARTICLE_ID where NAME is the author name and ARTICLE_ID is a WormBase article identifier. The ARTICLE_ID helps disambiguate between two authors who have the same name.

**Table 1:** Examples of WormBase links inserted in C. elegans articles

Entity Name	Entity Class	Link
pharynx	Anatomy_name	http://www.wormbase.org/db/get?name=pharynx;class=Anatomy_name

C06A6	Clone	http://www.wormbase.org/db/get?name=C06A6;class=Clone

ced-4	Gene	http://www.wormbase.org/db/get?name=ced-4;class=Gene

CED-4	Protein	http://www.wormbase.org/db/get?name=ced-4;class=Gene

Pun	Phenotype	http://www.wormbase.org/db/get?name=Pun;class=Phenotype

eDf11	Rearrangement	http://www.wormbase.org/db/get?name=eDf11;class=Rearrangement

WRM066aH05	Sequence	http://www.wormbase.org/db/get?name=WRM066aH05;class=Sequence

N2	Strain	http://www.wormbase.org/db/get?name=N2;class=Strain

otIs173	Transgene	http://www.wormbase.org/db/get?name=otIs173;class=Transgene

E189	Variation	http://www.wormbase.org/db/get?name=e189;class=Variation

Oliver Hobert	Person	http://www.wormbase.org/db/misc/person?name=Oliver%20Hobert;paper=WBPaper00036201

For *S. cerevisiae *yeast articles, SGD provides gene, protein, and variation entity lists. The URL pattern used is http://www.yeastgenome.org/cgi-bin/locus.fpl?dbid=SGDID, where SGDID is the SGD identification for the entity.

For *D. melanogaster *articles, FlyBase provides entities from five different classes: genes, alleles, aberrations, transgenic transposons and transposon insertions. The URL pattern used is http://flybase.org/reports/FBID.html, where FBID is the FlyBase identification for the entity. FlyBase suffers from the unique problem of having several common English words (for example: *for, we, a*) as gene names. Since we receive XML formatted files from the journal publisher, the policy is to link an entity only if it is italicized i.e. within italic tags in the XML.

### Database curators

After the paper has gone through the automated linking script, a link to the marked up paper is sent to the database curator for a manual quality control (QC) step to ensure all appropriate links are made. QC curators use an html viewer to manually assess each link for accuracy. The curator also searches the paper to make sure any new entity has been identified and linked. Additions, deletions and any corrections to the links in the XML file are done using a text editor or an XML editor.

### Ensuring stable URLs

An important requirement for this project is long-term stability of the database URLs. The databases need to be dedicated in maintaining the resource pages for the long term. To help the databases check whether the URLs inserted in the articles are live, a table of all the database links is maintained for each article, after all the manual QC steps are completed. Database curators periodically check the URLs to make sure the links are live.

## Hyperlinking pipeline set-up

**Figure 2:**
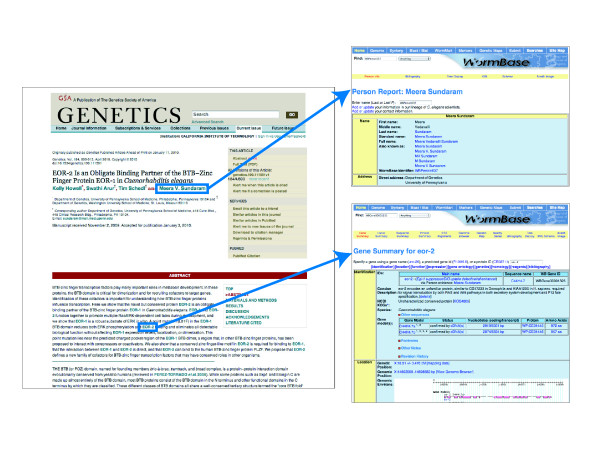
**An interactive *C. elegans *article with links to WormBase**. An entity-linked article with links to WormBase web pages, along with examples of the corresponding WormBase web pages

The journal GENETICS is a publication of the Genetics Society of America focused on research of inheritance in all organisms, including humans. We developed a hyperlinking pipeline to link entities in primary research articles focused on *Caenorhabditis elegans *published in GENETICS journal to relevant web pages in WormBase (Figure 2). This pipeline is a collaboration between the author, the journal, the journal services and the database. Our pipeline requires five elements:

1. A list of classes and named entities from the database.

2. A database URL pattern for generating a URL for each class.

3. Source file for the journal article, an XML provided by Dartmouth Journal Services (the journal publisher working with GENETICS).

4. A database curator for manual quality control.

5. A method of ensuring URL stability.

We initiated the project linking eight classes of entities (genes, proteins, variations, rearrangements, strains, transgenes, clones and sequences). As the project progressed we added three more data classes (anatomy term, author and phenotype). As of Dec 2010, 49 *C. elegans *articles have been processed through this pipeline; the first article published with online and PDF embedded hyperlinks to WormBase appeared in the Sept. 2009 issue of GENETICS [8]. Four of these 49 articles did not have any entities to link as they were either not a primary research article or they dealt with theoretical models of biological systems and did not mention any one gene or entity specifically.

The lexicon containing all entities used for linking is kept up to date through daily automated queries to local and site-wide WormBase databases for most of these entity classes. The sequence class, which is not updated daily, represents clone names from large scale experiments that do not change frequently. The other class that is not updated daily is the phenotype class, which represents three-letter short names adopted by researchers for ease of discussing somewhat complicated characteristics of their mutants. We are currently working out a way to update this entity class automatically as well.

### Authors' contributions

In addition to daily cron jobs, entity lists can be updated during this pipeline through a webform sent to authors (Figure 1) by GENETICS editors upon acceptance of the paper. WormBase provides the web form for authors to declare entities they have discovered or described in their paper, which would not yet exist in the database. A link to this web form is emailed to authors as soon as their article is accepted for publication. Authors have an average of 17 days (based on the first 25 articles we processed) to submit the form; this time corresponds to the time between receiving notification of their paper being accepted, which is accompanied by the link to the web form and the time a copy-edited XML version of their paper is sent to WormBase for linking.

The linking program automatically links data entered in this form to WormBase pages, so by choosing to participate through the author form, the authors are tasked with two responsibilities - first, to declare objects that do not exist in the database and second, to enter that data in a format that is machine readable. As the linking script takes up to 15 minutes to run (limited only by computer load), if an author has correctly declared all new entities, there is no need to run the script again. If the authors have not declared any new entities, we have the choice of running the script after an initial QC step or to make all corrections to the XML manually, which takes less than two minutes per entity per document. Thus author participation can save 15 minutes of waiting time for the linking script to run or a minimal amount of time to add the links to the document manually.

Of the 45 articles processed that contained entities for linking, authors of 17 articles submitted the web form to declare entities that had not yet been entered into WormBase. Manual quality control actually discovered 29 articles that had new entities, so even though author contributions are helpful, they are not entirely thorough.

Authors have another chance to comment on the linking that happens to their paper in this pipeline. After the article has gone through the automatic linking and manual QC step, the XML is made available to DJS via FTP to their server. DJS processes the final marked up XML into html and PDF formats. The hyperlinked PDF is sent to WormBase for final link proofing and then sent to the author. In three cases, the authors have requested links be corrected. In two cases authors caught errors we had missed and requested proper links. In the third case, an author requested corrections to the author name links. So author participation at this stage is very helpful and should be encouraged.

### Necessity and benefits of manual quality control

As mentioned above, the manual QC step can be used to deal with new objects that do not exist in WormBase. Whether or not the author declares these entities, we can identify them and incorporate them into the database within the next database release or soon thereafter. Our author first pass form also serves the dual purpose of alerting WormBase curators that the paper being processed contains a data type relevant to their curation pipeline.

In addition to identifying new entities missed by authors, we use the manual QC step to resolve ambiguous links as well as to correct linking errors incurred by author typos or XML file processing. Since we use an automated linking script that identifies entities in a paper based on a list of known entities, if an entity name is used more than once for distinct objects in the database, the script will potentially link the entity name to the wrong page; for example, the entity *AB1*, in the *C. elegans *literature, is both a clone and a strain name. We have found and corrected ambiguous links in 15 papers so far via the manual QC step.

Errors from ambiguous entity names can be accurately resolved only through human oversight. For *C. elegans *articles, little effort is required for resolving ambiguities because the number of ambiguous entities within the *C. elegans *field is low; this aspect of *C. elegans *research is a benefit of the simple and strict nomenclature rules that were put in place at the onset of adopting *C. elegans *as a model organism for genetic dissection [9]. On the other hand, ambiguities across databases may occur in *C. elegans *papers; for example, HID-1 is the name of a protein in *C. elegans *and in mouse [10]. In this case, WormBase links to HID-1 were selectively deleted when the authors were referencing the mouse HID-1.

An unintentional advantage of this pipeline is that our manual QC step also resolves author typos and XML formatting errors that occur during the document processing step at DJS; these types of errors result in wrong links or no link, so need to be dealt with in order to have the proper links made. We discovered these types of errors in at least 17 papers. We deal with these errors by alerting DJS who then contacts the author to sort out how to fix the typo. Because we are the main portal for *C. elegans *research and we are trained to spot details concerning variations, genes, transgenes etc., spotting these errors and understanding what the correction should be is not generally difficult. In most cases, we can go ahead and make the correct link to the typo and leave the author to request journal services to correct the text after we are through with the linking, so there is usually no waiting time involved in these situations.

On average it takes a WormBase curator 40 minutes to perform manual QC on a paper. The time required depends on many factors, the most important of which is the number of entity links generated in each paper. For example in five recent *C. elegans *articles, an average of 79 different unique URLs are generated for each article, many of which are propagated throughout the paper resulting in an average of 424 links per article; time is spent on ensuring that the initial URL is correct and that it is appropriate for the context.

## Linking SGD articles

Unlike *C. elegans *articles, SGD articles require a stable and unique SGDID for each gene for correct linking. Since a few primary yeast gene symbols have changed or switched with another gene, manual quality control is needed to identify the correct SGDID for these ambiguous gene symbols, as well as ambiguous reagents. However, since the number of ambiguous names is very low, the manual effort is minimal. In the most recent twenty papers we processed, we found an average of one ambiguous entity in every two papers.

## Linking FlyBase articles

We have set up a pipeline for linking *D. melanogaster *articles to FlyBase. As noted earlier, because of the non-standard FlyBase nomenclature, only entities that are italicized are linked to FlyBase. This policy eliminates most of the false positives that occur from gene names that are also common English words. (This policy introduces false negatives when a gene name is not italicized, but the recall reduction was well worth the precision enhancement.) Nevertheless we noticed that this policy might still give rise to a few false positives for very short gene names (like *N, P, a*) since some of them are used for mathematical symbols, which are also italicized. Currently these false positives are unlinked at the manual QC step.

## Linking multi-database articles

Having progressed with linking articles to SGD and Flybase, the next step of the project is linking entities from all databases should they occur together in the same paper. We have already carried out this task with the linking of both WormBase and SGD entities in Maduzia et al., 2010. [11]. For this article, the source XML was first run through the WormBase linking script. The output of this script was then fed as input to the SGD linking script. Since there were no nomenclature clashes between WormBase and SGD entities in this paper, it was easy to process this article, and a minimal amount of QC was necessary beyond the normal QC for each database's entities.

## Discussion

Over the past five years, we have seen a push by databases to streamline data extraction from journal articles through a variety of methods including the development of text-mining tools, the development of stand-alone user interfaces that can mine and hyperlink journal articles, and through working directly with journals to request authors to provide a minimum amount of information to the database [1,12-16]. Authors are valuable participants in our pipeline even though our experience with author participation demonstrates they are not completely reliable in providing the data we request. Our experience supports the results of the FEBS Letters experiment and the BioCreative II.5 challenge [12], in which author performance was determined to be relatively low, and further, their participation did not save trained curators any significant time in the identification and extraction of relevant data. We also agree with the conclusion of the BioCreative II.5 assessment that author participation is useful when combined with database-generated annotations, both human and machine. Regardless, we think that it is still very important to involve the authors in this pipeline. Ultimately, we hope that the more frequently authors are asked to participate in database curation efforts the more such participation becomes a standard part of having their work published.

We have a narrow goal in asking for author participation in this pipeline, that is, for them to help us link objects that do not exist in the database lexicon repository, and thus our database. We have taken advantage of this author submission pipeline to feed into a literature triage pipeline whereby data-type curators who are not part of this markup pipeline are alerted when there are new objects present in the paper, which will need curating. This latter use of author submissions is not a primary focus of this project, but it does provide valuable communication from which the author and our database benefits. Any level of participation from authors is beneficial to us; we are fortunate that we have had reasonable author participation.

### The proper balance between automation and manual efforts

A recent article by Atwood et al. [17] presented a nice overview of the different hyperlinking tools currently available. While fully automated hyperlinking tools can provide instantaneous links, be portable enough to use on any online html page and can form links to any source, they are at a disadvantage when it comes to ensuring accuracy. By using a manual QC step we can selectively unlink ambiguous terms, ensuring that the reader is taken to the correct webpage. One suggested solution to resolve these ambiguities is to rely on user feedback and employ the reader to correct links, which is in use by Utopia Documents [14] and *Reflect *[1] (relayed to us by one of anonymous reviewers). While this may prove an optimal solution for these fully automated tools, as we are starting from the point of the actual database that is being linked to, we might not benefit as much as these other projects. However, we are open to modifications of our pipeline that would increase our efficiency. As the most time consuming steps of this pipeline are the manual QC and XML editing we are actively developing tools to cut down on these steps. For example, we have created an interface that allows the curator to more easily view the marked up html as well as a list of the entities and links.

### Benefits to the community

We established a manual QC step to resolve ambiguous or erroneous links that occur with automated linking. Automated tools cannot distinguish one entity from another entity with the same name, even if the entities are, for example, genes in entirely different species. Because of the well-established nomenclature in the *C. elegans *field, ambiguity is low for most of the entity classes. As we begin to link terms in classes that use a more familiar term such as the anatomy class, we have noted a rise in the number of links to resolve.

We use our QC step to enrich our database by identifying entities missing from our database. We also can take advantage of the QC step to link synonyms of terms to the appropriate community-approved name. These synonyms could represent jargon terms or author assigned names that did not follow community-based nomenclature rules when first adopted. For example, a researcher may have cloned a gene and assigned a name to it without first finding out if the gene name conflicts with a pre-existing class of genes soon to be published. In such cases, a link between the gene name used by the author can be made to the correct sequence page at the database, offsetting any confusion should the gene name get changed after the article is published.

By far the most important duty of this step is to resolve ambiguous links; however we have taken advantage of this step to feed important information back to the authors and journal as well as to enhance our own database. For example, we use the author first pass form to capture new entities that don't exist in our database. Combined with the manual QC step, we are able incorporate data in our database before the paper is published. In addition, our manual QC step has identified entities that had been discovered years ago but had not been entered into the database yet, which occurred for two papers.

Finally, our manual QC step has proved beneficial to the authors and journal as we have been able to catch typos and XML formatting errors that were missed by the authors and copy-editors.

### Resolving ambiguities

The hardest hurdle in all of the hyperlinking efforts to date is resolving which species an ambiguous entity belongs to (see [18] for example). One automated method, Linneaus, has been developed to tackle the problem of identifying all gene names belonging to all species in an article without any prior knowledge of which species are discussed [19,20]. However, our problem differs from others in that we have an advantage of knowing the different species discussed in an article *a priori *from the journal publisher and the authors. Hence the problem of species identification itself is not a major challenge we face. In addition, as noted, authors usually specify the species name near the entity should there be any ambiguity [20]. For example, authors may use abbreviations (*Sc *for *S. cerevisiae, Sp *for *Schizosaccharomyces pombe*) before the entity name for disambiguation, which could be identified before linking is started. Finally, since we expand our pipeline one MOD at a time and work closely with MOD curators, identifying unique styles and conventions of writing scientific articles for each species could be captured and used for automatic disambiguation. While proximity of the species name to the entity does help in ambiguity resolution in most cases, such a heuristic approach may not work for all cases because of complexities in natural language texts. Ultimately, the manual QC step still remains the best way to identify and correct any errors arising out of automatic methods.

We are planning on linking articles from more model organisms and are looking for other databases and journals to actively participate in this project.

## Authors' contributions

AR developed, maintains and runs the linking software and the software for obtaining  the entities from the biological databases. TS initiated the project, provided the  workflow, did initial manual quality control and oversees the project. KY does the  manual quality control for WormBase and provides feedback to correct the linking  software and the pipeline, and designed the author form for declaring new entities.  JC set up the author form, integrated author data to forms used by WormBase  curators and provides access to WormBase data. SH runs the source XML  composition software. LO, SF send article source files to databases and receive the  returned, linked article, and communicate with the authors at the proof stage. TDC  helped to initiate and oversees the project. RI obtains a WormBase paper ID for each  new article published by Genetics and sends authors the new author form.  MSS does manual QC for SGD. JMC initiated the project at SGD, provided access to  SGD data and oversees the project for SGD. RS does manual QC for FlyBase. SJM initiated the project at FlyBasae, provided access to FlyBase data and oversees the project for FlyBase. PWS oversees the project at WormBase,  and helps with QC. HMM provided the textpresso software used as part of the linking  software, and also helps run the software. All the authors read and approved the  final manuscript.  
